# 固相萃取-超高效液相色谱-串联质谱法测定人尿中苯氧乙酸除草剂和有机磷、拟除虫菊酯农药代谢物

**DOI:** 10.3724/SP.J.1123.2022.05005

**Published:** 2023-03-08

**Authors:** Xu ZHANG, Linxue HAN, Tian QIU, Xiaojian HU, Ying ZHU, Yanwei YANG

**Affiliations:** 中国疾病预防控制中心环境与人群健康重点实验室, 中国疾病预防控制中心环境与健康相关产品安全所, 北京 100021; China CDC Key Laboratory of Environment and Population Health, National Institute of Environmental Health, Chinese Center for Disease Control and Prevention, Beijing 100021, China

**Keywords:** 固相萃取, 超高效液相色谱-串联质谱, 农药, 代谢物, 尿液, 内暴露, 生物监测, solid phase extraction (SPE), ultra-performance liquid chromatography-tandem mass spectrometry (UPLC-MS/MS), pesticides, metabolites, urine, internal exposure, biomonitoring

## Abstract

基于96孔固相萃取-超高效液相色谱-串联质谱法,建立了人尿中2种苯氧乙酸除草剂、2种有机磷农药代谢物和4种拟除虫菊酯农药代谢物的测定方法。通过对液相色谱条件、质谱条件和样品前处理过程的系统优化,实现了在16 min内对8种目标分析物的分析测定。具体方法:1 mL尿液经*β*-葡萄糖醛酸酶酶解过夜,Oasis HLB 96孔固相萃取进行目标分析物的提取净化,甲醇洗脱;以0.1%(体积分数)乙酸乙腈和0.1%(体积分数)乙酸水作为流动相,Acquity BEH C_18_作为分析柱进行色谱分离;负离子电喷雾(ESI^-^)多反应监测(MRM)模式下检测目标化合物,同位素内标法定量。2,4-二氯苯氧乙酸(2,4-D)、2,4,5-三氯苯氧乙酸(2,4,5-T)2种苯氧乙酸除草剂和3-苯氧基苯甲酸(3-PBA)、4-氟-3-苯氧基苯甲酸(4F-3PBA)、反式二氯乙烯基二甲基环丙烷羧酸(*trans*-DCCA)3种拟除虫菊酯农药代谢物在0.1~100 μg/L内、对硝基苯酚(PNP)、3,5,6-三氯-2-吡啶酚(TCPY)2种有机磷农药代谢物、顺式二氯乙烯基二甲基环丙烷羧酸(*cis*-DCCA) 1种拟除虫菊酯代谢物在0.2~100 μg/L内线性关系良好,相关系数均大于0.9993;方法检出限为0.02~0.07 μg/L,方法定量限为0.08~0.2 μg/L;低、中、高3个水平下的加标回收率为91.1%~110.5%,日内精密度为2.9%~7.8%,日间精密度为6.2%~10%。应用该方法测定了214份尿液样本。结果显示除2,4,5-T外,其余7种目标分析物均有检出。TCPY、PNP、3-PBA、4F-3PBA、*trans*-DCCA、*cis*-DCCA、2,4-D的检出率为2.8%~99.1%。检出浓度(中位值)由高到低分别是2.0 μg/L(TCPY)、1.8 μg/L(PNP)、0.99 μg/L(*trans*-DCCA)、0.81 μg/L(3-PBA)、0.44 μg/L(*cis*-DCCA)、0.35 μg/L(2,4-D)和未检出(4F-3PBA)。该方法操作简便,定量准确,灵敏度高,每批次可完成96个样品测定,适用于人尿中多种农药及农药代谢物的批量分析测定。

## Abstract (en)

Pesticides are widely used in most agricultural areas to protect food crops but adversely affect ecosystems and human beings. Pesticides have attracted great public concern due to their toxic properties and ubiquitous occurrence in the environment. China is one of the largest users and producers of pesticides globally. However, limited data are available on pesticide exposure in humans, which warrants a method for quantification of pesticides in human samples. In the present study, we validated and developed a comprehensive and sensitive method for the quantification of two phenoxyacetic herbicides, two metabolites of organophosphorus pesticides and four metabolites of pyrethroid pesticides in human urine using 96-well plate solid phase extraction (SPE) coupled with ultra-performance liquid chromatography-tandem mass spectrometry (UPLC-MS/MS). For this purpose, a systematic optimization of the chromatographic separation conditions and MS/MS parameters was conducted. Six solvents were optimized for the extraction and clean-up of human urine samples. The targeted compounds in the human urine samples were well separated within 16 min in one analytical run. A 1 mL aliquot of human urine sample was mixed with 0.5 mL sodium acetate buffer (0.2 mol/L) and hydrolyzed by *β*-glucuronidase enzyme at 37 ℃ overnight. The eight targeted analytes were extracted and cleaned using an Oasis HLB 96-well solid phase plate and eluted with methanol. The separation of the eight target analytes was performed on a UPLC Acquity BEH C_18_ column (150 mm×2.1 mm, 1.7 μm) with gradient elution using 0.1% (v/v) acetic acid in acetonitrile and 0.1% (v/v) acetic acid in water. The analytes were identified using the multiple reaction monitoring (MRM) mode under negative electrospray ionization (ESI^-^) and quantified by isotope-labelled analogs. Para-nitrophenol (PNP), 3,5,6-tricholor-2-pyridinol (TCPY) and *cis*-dichlorovinyl-dimethylcyclopropane carboxylic acid (*cis*-DCCA) exhibited good linearities ranging from 0.2 to 100 μg/L, and 3-phenoxy benzoic acid (3-PBA), 4-fluoro-3-phenoxy benzoic acid (4F-3PBA), 2,4-dicholorphenoxyacetic acid (2,4-D), *trans*-dichlorovinyl-dimethylcyclopropane carboxylic acid (*trans*-DCCA) and 2,4,5-tricholorphenoxyacetic acid (2,4,5-T) showed linearity ranging from 0.1 to 100 μg/L with correlation coefficients all above 0.9993. Method detection limits (MDLs) and method quantification limits (MQLs) of targeted compounds were in the range of 0.02 to 0.07 μg/L and 0.08 to 0.2 μg/L, respectively. The spiked recoveries of target compounds at three levels of 0.5, 5 and 40 μg/L were 91.1% to 110.5%. The inter- and intra-day precisions of targeted analytes were 2.9% to 7.8% and 6.2% to 10%, respectively. This method was applied to the analysis of 214 human urine samples across China. The results showed that all the targeted analytes, except 2,4,5-T, were detected in human urine. The detection rates of TCPY, PNP, 3-PBA, 4F-3PBA, *trans*-DCCA, *cis*-DCCA, and 2,4-D were 98.1%, 99.1%, 94.4%, 2.80%, 99.1%, 63.1% and 94.4%, respectively. The median concentration of targeted analytes in a decreasing order were: 2.0 μg/L (TCPY), 1.8 μg/L (PNP), 0.99 μg/L (*trans*-DCCA), 0.81 μg/L (3-PBA), 0.44 μg/L (*cis*-DCCA), 0.35 μg/L (2,4-D) and below MDLs (4F-3PBA ). For the first time, we developed a method to extract and purify specific biomarkers of pesticides from human samples based on offline 96-well SPE. This method has the advantages of simple operation, high sensitivity, and high accuracy. Moreover, up to 96 human urine samples were analyzed in one batch. It is suitable for the determination of eight specific pesticides and their metabolites in large sample sizes.

农药是农业生产中的重要生产要素,在提高农作物产量和保障粮食安全中发挥重要作用。近几十年,农药在全球范围内的生产和使用呈现增长趋势^[1⇓-3]^。1995~2020年,我国各类农药的年总产量从41.65万吨增长到214.8万吨^[4]^,成为全球最大的农药生产国和消费国^[5]^。农药的大规模使用已对我国生态环境造成潜在威胁^[6]^。空气^[7]^、地表水^[8]^、地下水^[9]^、土壤^[10]^等环境介质和果蔬^[11]^等农产品中均可检出农药残留。其中华东地区城市空气中毒死蜱和拟除虫菊酯总含量为150~3816 pg/m^3[7]^;华中地区地表水、地下水中有机磷农药总含量分别为81.5~225.1 ng/L和17.6~321.8 ng/L^[12]^。农药暴露可造成神经系统^[13,14]^、呼吸系统^[15]^和内分泌系统^[16]^损伤,还能诱导肿瘤发生^[17]^,其暴露也会对孕妇及儿童等环境易感人群产生健康影响^[18]^。针对农药开展的暴露评估已成为环境健康领域的研究热点。

除有机氯等持久性农药外,大部分在售农药在人体内无明显蓄积,数小时或数日内可通过肾脏随尿液排出体外^[19]^。因此,测定尿中农药或代谢物的浓度水平,可在一定程度上反映其暴露数据。美国、加拿大等国家已针对其本国居民开展了多次农药暴露监测工作,监测指标涉及乐果、呋喃酚、2-异丙基-6-甲基-4-吡啶醇、4-氟-3-苯氧基苯甲酸(4F-3PBA,氯氟氰菊酯代谢物)、3-苯氧基苯甲酸(3-PBA,氯菊酯和氯氰菊酯代谢物)、顺式二氯乙烯基二甲基环丙烷羧酸(*cis*-DCCA,溴氰菊酯代谢物)、反式二氯乙烯基二甲基环丙烷羧酸(*trans*-DCCA,氯菊酯和氯氰菊酯代谢物)、2,4-二氯苯氧乙酸(2,4-D)、2,4,5-三氯苯氧乙酸(2,4,5-T)等50余种农药类生物标志物;采用的分析方法包括高效液相色谱-串联质谱法(HPLC-MS/MS)、在线固相萃取-液相色谱-串联质谱法(Online-SPE-LC-MS/MS)和气相色谱-质谱法(GC-MS)等。我国于2017年启动国家人体生物监测工作,已将农药作为一类重要的监测指标^[20]^。因此,亟需建立准确、稳定和快速的分析方法,以满足大样本量的分析测定工作。Mark等^[21]^利用全自动固相萃取处理尿液样本,HPLC-MS/MS法测定尿中对硝基苯酚(PNP,对硫磷代谢物)、3,5,6-三氯-2-吡啶酚(TCPY,毒死蜱代谢物)等12种特异性的农药标志物。该法前处理通量高,测定物质种类多,但其在前处理过程中使用的丙酮溶剂在我国属于管制品,不易获得,导致方法使用受限。加拿大生物监测项目采用液液萃取-柱前衍生的方法进行尿液前处理,再利用UPLC-MS/MS和GC-MS测定TCPY及3-PBA等4种拟除虫菊酯代谢物^[22]^。该法存在操作繁琐、处理效率低、有机溶剂用量大等缺点,对环境不够友好且不适用于大样本的检测。

本工作建立了人尿中2种苯氧乙酸类除草剂(2,4-D、2,4,5-T)、2种有机磷类农药代谢物(PNP、TCPY)和4种拟除虫菊酯类农药代谢物(3-PBA、4F-3PBA、*cis*-DCCA、*trans*-DCCA)共8种农药及农药代谢物的固相萃取-液相色谱-串联质谱测定方法。该法采用96孔离线固相萃取处理尿液样本,具有操作简单、测定结果准确、稳定性好、样品处理通量高的优点,适用于尿液样本中这8种农药及农药代谢物的快速批量测定。

## 1 实验部分

### 1.1 仪器、试剂与材料

AB SCIEX 6500^+^三重四极杆质谱仪(美国AB公司); I-Class超高效液相色谱(美国沃特世公司); 2K-15高速离心机(美国SIGMA公司);恒温水浴摇床(莱伯泰科仪器股份有限公司); 96孔固相萃取装置(美国沃特世公司); MPC-2000离心机(北京鼎昊科技有限公司); IQ7005超纯水机(美国Milli-Q公司); NDK200-1A氮吹仪(杭州米欧仪器公司)。

农药及农药代谢物标准溶液:PNP、TCPY、2,4-D、2,4,5-T单标溶液购自Accu-standard公司,质量浓度均为100 μg/mL,纯度>97%; 4F-3PBA购自Ehrenstorfer公司,质量浓度为100 μg/mL,纯度>98%; 3-PBA、*cis*-DCCA、*trans*-DCCA和8种目标分析物的同位素内标单标溶液(^13^C_6_-PNP、^13^C_3_-TCPY、^13^C_6_-3-PBA、^13^C_6_-4F-3PBA、^13^C_2_-D_1_-*cis*-DCCA、^13^C_2_-D_1_-*trans*-DCCA、^13^C_6_-2,4-D、^13^C_6_-2,4,5-T)购自Cambridge Isotope Laboratories公司,质量浓度均为100 μg/mL,纯度>97%。

甲醇(色谱级)购自德国Merck公司;乙腈(色谱级)、无水乙酸钠(色谱级)和*β*-葡萄糖醛酸酶(酶活力>85000 U/mL)购自美国SIGMA公司;乙酸(色谱级)购自美国TIEDA公司;96孔Oasis HLB固相萃取柱(30 mg)购自美国沃特世公司。

### 1.2 实验条件

#### 1.2.1 色谱条件

色谱柱:Waters Acquity BEH C_18_(100 mm×2.1 mm, 1.7 μm);流动相A: 0.1%(体积分数)乙酸水;流动相B: 0.1%(体积分数)乙酸乙腈;进样量:10 μL;柱温:40 ℃。梯度洗脱:0~1.0 min, 95%A; 1.0~2.0 min, 95%A~80%A; 2.0~4.0 min,保持80%A; 4.0~5.0 min, 80%A~60%A; 5.0~7.0 min,保持60%A; 7.0~8.0 min, 60%A~50%A; 8.0~10.0 min,保持50%A; 10.0~10.5 min, 50%A~0A; 10.5~12.5 min,保持0A; 12.5~13.0 min, 0A~95%A; 13.0~16.0 min,保持95%A。

#### 1.2.2 质谱条件

离子源:电喷雾电离源(ESI);电离方式:负电离;扫描模式:多反应监测(MRM)模式;碰撞气(CAD): High;气帘气(CUR): 207 kPa;雾化气(GS1): 345 kPa;加热气(GS2): 414 kPa;喷雾电压(IS): -4500 V;离子源温度(TEM): 350 ℃;入口电压(EP): -10 V;扫描时间:30 ms。8种目标分析物及内标的其他质谱参数见表1。

**表1:** 8种目标物及其同位素内标的质谱参数

Compound	Retention time/min	Parent ion (m/z)	Daughter ion (m/z)	DP/V	CE/eV
Para-nitrophenol (PNP)	5.80	138.0	108.0^*^	-40	-24
			92.1	-40	-30
3,5,6-Tricholor-2-pyridinol (TCPY)	7.71	196.0	35.1^*^	-30	-44
		198.0	35.1	-30	-40
3-Phenoxy benzoic acid (3-PBA)	8.95	213.1	93.0^*^	-30	-26
			65.1	-30	-70
4-Fluoro-3-phenoxy benzoic acid (4F-3PBA)	9.05	231.1	93.1^*^	-30	-28
			65.1	-30	-75
cis-Dichlorovinyl-dimethylcyclopropane	9.32	206.9	35.1^*^	-20	-35
carboxylic acid (cis-DCCA)		208.9	35.1	-20	-35
trans-Dichlorovinyl-dimethylcyclopropane	8.91	207.1	35.2^*^	-20	-40
carboxylic acid (trans-DCCA)		209.1	35.2	-30	-40
2,4-Dicholorphenoxyacetic acid (2,4-D)	7.30	219.0	161.1^*^	-30	-18
			125.0	-30	-36
2,4,5-Tricholorphenoxyacetic acid (2,4,5-T)	8.54	253.1	195.0^*^	-20	-19
			159.0	-20	-39
^13^C_6_-PNP	5.79	144.1	114.1	-20	-10
^13^C_3_-TCPY	7.70	201.1	35.1	-50	-40
^13^C_6_-3-PBA	8.94	219.1	99.1	-25	-27
^13^C_6_-4F-3PBA	9.04	236.9	99.1	-30	-30
^13^C_2_-D_1_-cis-DCCA	9.30	210.2	35.1	-30	-30
^13^C_2_-D_1_-trans-DCCA	8.89	210.1	35.1	-40	-38
^13^C_6_-2,4-D	7.29	224.8	167.0	-30	-17
^13^C_6_-2,4,5-T	8.53	260.6	203.0	-40	-15

* Quantitative ion. DP: declustering potential; CE: collision energy.

### 1.3 溶液配制

混合标准储备液(4 mg/L):用移液管准确移取8种目标分析物单标溶液各1.0 mL至25 mL容量瓶中,用乙腈定容;将定容好的混合标准储备液放置在4 ℃下溶解12 h,再将混合标准储备液分装至2 mL安瓿瓶中,封口后于4 ℃保存。

混合同位素内标储备液(4 mg/L):按混合标准储备液配制方法配制混合同位素内标储备液。使用时,将混合同位素内标储备液用纯水稀释成100 μg/L的混合内标工作液使用。

系列标准溶液配制:用乙腈将混合标准储备液稀释至质量浓度为1000、100和10 μg/L的混合标准工作液;吸取适量混合标准工作液,配制成0.1、0.5、1、2.5、5、10、25、50、100 μg/L的系列标准溶液,内标添加量为20 μg/L。

### 1.4 样品前处理

将尿液样本平衡至室温,涡旋混匀后取1 mL尿液到5 mL聚丙烯离心管中。向离心管中加入500 μL乙酸钠溶液(0.2 mol/L)、100 μL混合内标工作液和15 μL *β*-葡萄糖醛酸酶,混匀后于37 ℃酶解过夜。酶解后取出样品降至室温,加入20 μL乙酸,振荡,4500 r/min离心10 min,取上清液进行固相萃取。

依次用1 mL甲醇和1 mL水活化及平衡每个固相萃取孔;在一定压力条件下进行样品上样(1滴/s)。上样完成后,每孔用1 mL 25%(体积分数)甲醇水溶液快速淋洗,空气抽干5 min,用1 mL甲醇分两次洗脱(每次0.5 mL)。洗脱液收集于96孔收集板中,氮吹浓缩至约200 μL,加入50 μL纯水作为保护剂。继续氮吹至50 μL,加入450 μL 10%乙腈水(体积分数)进行复溶,4500 r/min离心10 min后上机测定。

### 1.5 质量保证与控制

为降低空白干扰,实验使用的玻璃器皿、枪头和离心管等实验耗材均为一次性使用。实验试剂为HPLC级或MS级试剂;塑料离心管采用聚丙烯材质,且在每次使用前均用纯水和甲醇分别润洗2遍;玻璃试管清洗干净后在450 ℃下烘烤4 h以上。

## 2 结果与讨论

### 2.1 质谱条件优化

将50 μg/L单标溶液注入质谱仪进行目标分析物的质谱参数优化。由于8种目标化合物的结构中均含有羟基,在ESI源下易失去H而形成[M-H]^-^的母离子。因此,本实验在负电离模式下确定化合物及其同位素内标的母离子信息。确定母离子后,再依次进行子离子扫描,选择丰度较高的2个特征碎片离子作为子离子,同时优化各特征离子对的碰撞能量和去簇电压。最后在MRM模式下对CAD、CUR、GS1、TEM等质谱参数进行优化,使各目标化合物的离子化效果均处于最佳状态。

### 2.2 液相色谱条件优化

#### 2.2.1 色谱柱

采用Waters Acquity UPLC BEH C_18_ (100 mm×2.1 mm, 1.7 μm)、Waters Xbridge C_18_ (100 mm×2.1 mm, 2.5 μm)、Waters CSH C_18_ (100 mm×2.1 mm, 1.7 μm)、Waters HSS T_3_ (100 mm×2.1 mm, 1.7 μm)和Waters BEH Phenyl(100 mm×2.1 mm, 1.7 μm)柱对8种目标分析物进行色谱分离。结果显示,以乙腈-水为流动相时,8种目标分析物在CSH C_18_、HSS T_3_和BEH Phenyl柱上的保留效果不理想。目标分析物经3种色谱柱分离后的质谱响应显著低于BEH C_18_和Xbridge C_18_柱。除此之外,使用HSS T_3_和BEH Phenyl柱进行分析时,其色谱图的基线明显偏高。对比BEH C_18_和Xbridge C_18_色谱柱的分离效果,发现BEH C_18_柱在兼顾灵敏度的条件下实现了*cis*-DCCA和*trans*-DCCA两种同分异构体的完全分离。因此,实验选择BEH C_18_柱作为分析柱。

#### 2.2.2 流动相

以BEH C_18_作为分析柱,在甲醇-水和乙腈-水两种流动相体系下优化洗脱梯度。结果显示,无论如何调节洗脱梯度,甲醇-水进行洗脱时均无法实现*cis*-DCCA和*trans*-DCCA的有效分离,两种目标物表现为重叠峰或驼峰。当以乙腈-水作为流动相时,可通过调整洗脱梯度实现两种物质的完全分离。

在流动相中加入有机酸能改善带负电荷的目标分析物在色谱柱上的保留效果^[19]^。因此,向流动相中加入不同体积分数的乙酸(0.05%、0.1%和0.2%)以确定最优流动相。结果显示,随着乙酸浓度增加,目标分析物的色谱峰变窄,色谱分离度提高。然而,在改善分离效率的同时,乙酸对多种目标分析物的电离产生了抑制,导致灵敏度降低。因此,综合考虑同分异构体的分离效果和方法的灵敏度,实验最终选择0.1%(体积分数)乙酸乙腈和0.1%(体积分数)乙酸水作为流动相。在此流动相条件下,8种目标分析物的提取离子色谱图见图1。

**图1:**
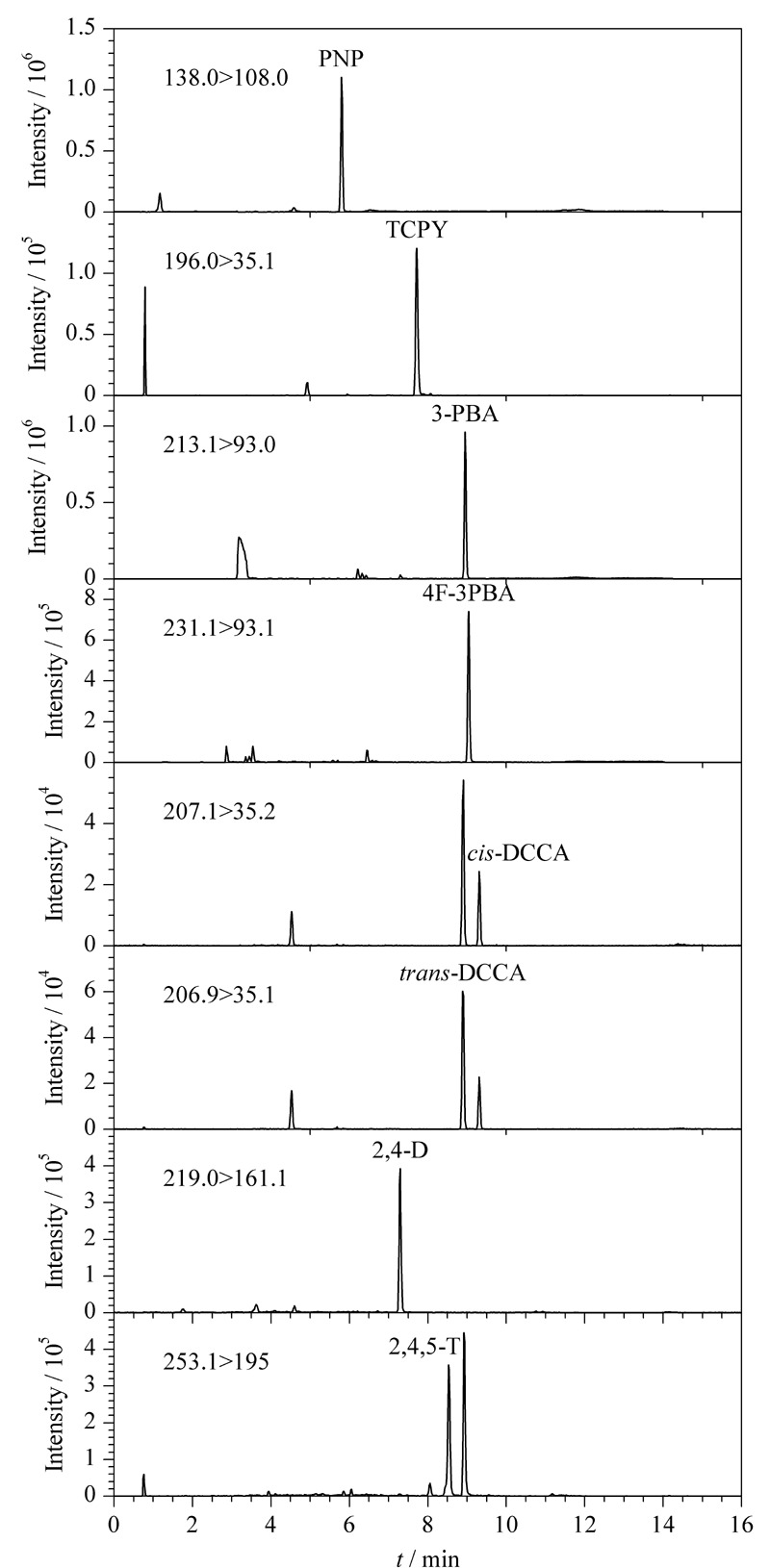
尿液中8种目标分析物的提取离子色谱图

### 2.3 前处理条件优化

#### 2.3.1 酶解条件优化

尿液中的目标分析物主要以葡萄糖醛酸酯和/或硫酸酯等结合物形式存在。因此,应先对样品进行酶解。Mark等^[21]^和Paramjit等^[23]^采用固体酶酶解过夜的方式处理尿液样本,酶活力分别>500 U/mL和>1000 U/mL。本课题组前期研究表明,来源相同(如全部来自罗曼蜗牛)的固体酶和液体酶,其酶解效率可能存在差异^[24]^。因此,方法比较了不同液体酶添加量(10、15和20 μL)的酶解效果。结果表明,添加10 μL液体酶时(酶活力>850 U/mL),尿中各目标分析物的测定值最低;添加量15 μL液体酶时(酶活力>1275 U/mL),尿中各物质的测定值进入平台期,与添加20 μL液体酶(酶活力>1700 U/mL)的测定结果一致。因此,本方法确定液体酶添加量为15 μL。在相同酶活力条件下,比较两个不同来源(来源于罗曼蜗牛和大肠杆菌)液体酶的酶解效果。结果表明,罗曼蜗牛液体酶对PNP和2,4-D两类物质的酶解效率更高,两类物质的测定值比大肠杆菌液体酶酶解后的测定值高1倍。因此,方法选择罗曼蜗牛*β*-葡萄糖醛酸酶进行酶解,添加量为15 μL(酶活力>1275 U/mL)。

#### 2.3.2 上样条件

对样品进行适度酸化能增加目标分析物在HLB柱上的富集和保留。向酶解完成的尿液中加入不同体积的乙酸(0、10、20、30 μL),比较目标分析物在不同乙酸添加条件下的质谱响应。峰面积越高,表明在该条件下有更多的目标物被富集在固相萃取柱上。结果显示,随着乙酸添加量增加,8种目标分析物的定量离子峰面积均呈现不同程度的增加,其中以2,4-D的增加最为显著。当乙酸添加量为20 μL时,目标分析物峰面积达到最高值。因此,本方法向酶解完成的样品中添加20 μL乙酸以提高目标分析物的灵敏度。

#### 2.3.3 淋洗条件

用不同体积分数(5%、10%、15%、20%、25%、30%)的甲醇水溶液淋洗固相萃取柱,以不同淋洗条件下各目标分析物的色谱峰面积大小来评估淋洗效果。结果显示,随着甲醇比例的增加,8种目标分析物的色谱峰面积均呈现增加趋势(增加幅度为10%~15%)。当甲醇体积分数为25%时,峰面积增加幅度最大。其原因是淋洗液中的甲醇比例升高导致淋洗液极性降低,低极性溶液在反相固相萃取柱上具有更大的淋洗强度。因此有更多的干扰物被淋洗,进而降低了基质对样品测定的干扰。而当淋洗液中甲醇比例升高至30%时,此时由于淋洗强度变强,部分目标化合物可能随干扰物一同被淋洗,从而导致目标物丢失。此外,实验向25%甲醇水溶液中分别添加0.5%、1%和2%(体积分数)的乙酸,以考察酸性条件对淋洗效果的影响。结果表明,8种目标分析物的峰面积在不同酸性环境淋洗条件下无明显差异。因此,实验选择25%甲醇水溶液作为淋洗溶剂。

#### 2.3.4 洗脱条件

向尿液中加入5 μg/L目标分析物,以甲醇、乙腈、甲醇-乙腈(1∶1, v/v)、甲醇-乙酸乙酯(1∶1, v/v)、乙腈-乙酸乙酯(1∶1, v/v)和丙酮作为洗脱溶剂,通过回收率比较6种洗脱溶剂洗脱效果。如图2所示,以甲醇和甲醇-乙腈(1∶1, v/v)作为洗脱溶剂时,8种目标分析物的回收率结果差异不明显,但优于其他4种洗脱溶剂。为获得较优洗脱溶剂,分别对6种洗脱条件下各目标分析物的基质效应(matrix effects)进行了初步评估。参考Li等^[25]^采用的单点浓度评估方法,分别向不同洗脱溶剂处理后的样品基质和纯溶剂基质中加入5 μg/L的目标分析物,以目标分析物在样品基质中的峰面积与相同目标分析物在纯溶剂中的峰面积的比值评估基质效应。当基质效应在80%~120%时为低基质效应;基质效应在50%~80%或120%~150%时为中基质效应;基质效应<50%或>150%时为高基质效应。结果如图3,以甲醇为洗脱溶剂时,所有目标分析物均表现为低基质效应或中基质效应;而以甲醇-乙腈(1∶1, v/v)作为洗脱剂时,PNP表现为高基质效应。因此,实验最终选择甲醇作为洗脱溶剂。

**图2:**
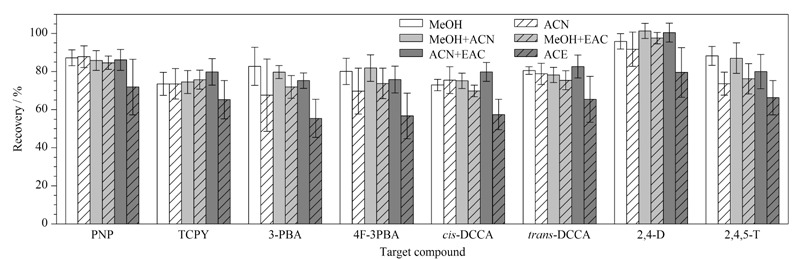
不同洗脱溶剂对8种目标分析物回收率的影响(*n*=3)

**图3:**
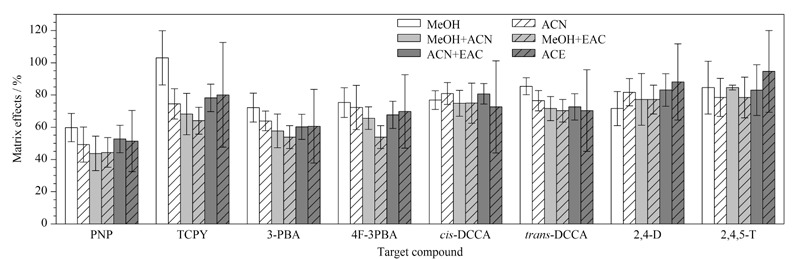
不同洗脱溶剂对8种目标分析物的基质效应(*n*=3)

使用2 mL甲醇分4次洗脱固相萃取柱(每次0.5 mL)。收集4次洗脱液并上机测定,以每次洗脱后目标分析物的峰面积占4次洗脱的总面积的百分比计算洗脱效率。结果如表2所示,完成第1次洗脱时,目标分析物的洗脱效率为90.3%~98.4%;继续进行第2次洗脱,仍有部分目标化合物被持续洗脱下来;在完成2次洗脱后,各目标分析物的洗脱效率已达到99%以上;此时再增加洗脱次数,对洗脱效率影响并不显著。因此,实验最终选择2次洗脱(每次0.5 mL)的方式洗脱目标化合物。

**表2:** 不同洗脱次数下8种目标分析物的洗脱效率

Compound	Elution efficiencies/%			
	1^st^	2^nd^	3^rd^	4^th^
PNP	95.8	3.64	0.38	0.18
TCPY	97.0	2.36	0.35	0.25
3-PBA	90.3	8.88	0.60	0.23
4F-3PBA	94.4	4.96	0.40	0.24
cis-DCCA	98.3	1.27	0.27	0.19
trans-DCCA	98.4	1.13	0.25	0.19
2,4-D	98.0	1.56	0.28	0.21
2,4,5-T	97.0	2.36	0.35	0.25

### 2.4 基质效应

样本中存在的复杂基质可能对目标物的分析过程产生干扰,从而影响方法的准确度和精密度。因此需要对方法的基质效应进行评估,以确定是否需要进行基质补偿或校正。采用文献报道的方法^[25,26]^,选用6个不同来源的尿液,按前处理方法进行样本处理后,以6个处理尿液为基质配制6个系列的质量浓度为0、1、2.5、5、10、25、50、100 μg/L的尿液基质标准溶液,同时配制相同浓度的系列甲醇基质标准溶液。以尿液基质标准曲线的斜率与甲醇基质标准曲线的斜率的比值来评估方法的基质效应。

基质效应评估结果如图4所示,对于PNP和*cis*-DCCA,有4个尿液基质表现为中基质效应,2个尿液基质表现为低基质效应;在3-PBA和2,4-D中,有3个尿液基质表现为中基质效应和3个尿液基质表现为低基质效应。上述结果表明在分析过程中,需要采取措施对上述目标分析物进行基质校正。同位素内标对8种物质的基质校正结果见表3。当加入同位素内标后,8种目标分析物的基质效应为94.1%~100%,均表现为低基质效应。因此,本方法采取同位素内标法进行定量分析。

**图4:**
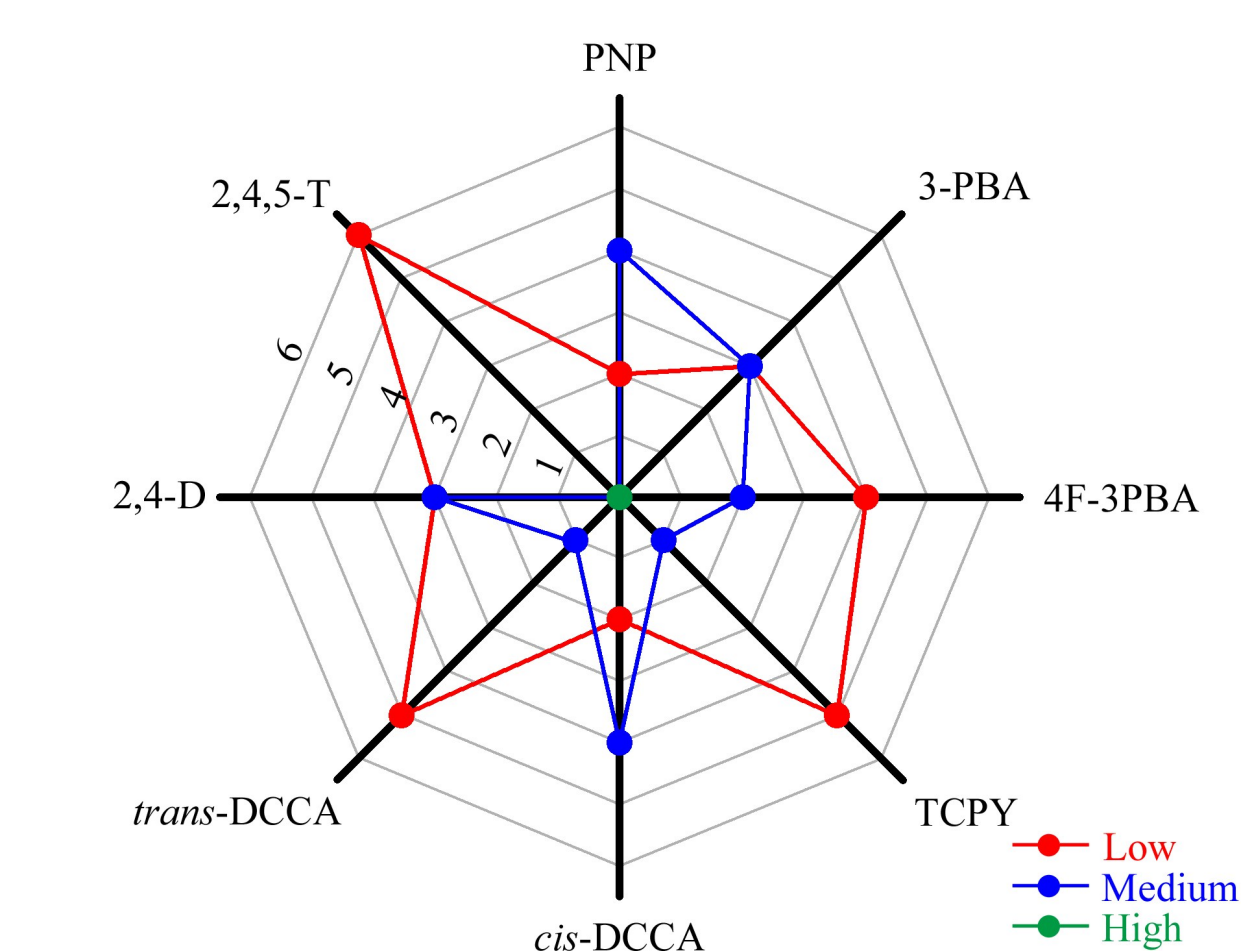
8种目标分析物在6个不同尿液基质中的基质效应

**表3:** 8种目标分析物的线性范围、相关系数、 方法检出限、方法定量限和基质效应

Compound	Linear range/ (μg/L)	r^2^	MDL/ (μg/L)	MQL/ (μg/L)	Matrix effect/%
PNP	0.2-100	0.9996	0.07	0.2	100
TCPY	0.2-100	0.9993	0.05	0.2	99.9
3-PBA	0.1-100	0.9993	0.03	0.1	98.1
4F-3PBA	0.1-100	0.9998	0.02	0.08	95.2
cis-DCCA	0.2-100	0.9995	0.06	0.2	97.4
trans-DCCA	0.1-100	0.9994	0.03	0.1	98.5
2,4-D	0.1-100	0.9994	0.03	0.1	94.1
2,4,5-T	0.1-100	0.9998	0.02	0.08	96.1

### 2.5 方法学评价

#### 2.5.1 线性范围、检出限与定量限

配制质量浓度为0.1、0.2、0.5、1、2、5、10、20、50、100 μg/L的系列标准溶液并进样分析,以目标物与相应内标的定量离子峰面积之比为纵坐标,样品中目标物的质量浓度为横坐标绘制标准曲线。8种目标物在各自的线性范围内相关系数(*r*^2^)均≥0.9993。检出限和定量限根据美国环保署(United States Environmental Protection Agency, USEPA)检出限测定程序第2版(EPA 821-R-16-006规范文件)计算^[27]^。2,4-D、TCPY、PNP存在过程空白干扰,以7次过程空白的均值加3倍标准偏差为检出限,7次过程空白均值加10倍标准偏差为定量限。其余5种目标分析物,选择测定值较低的尿液样本,用纯水稀释5倍制成空白尿液。向此空白尿液基质进行0.1 μg/L加标(*n*=7),以测定值的3倍标准偏差计算方法检出限(MDL)、10倍标准偏差计算方法定量限(MQL)。8种目标分析物的线性范围、相关系数、MDL、MQL等数据见表3。

#### 2.5.2 回收率与精密度

以6个实际尿液样本进行低(0.5 μg/L)、中(5 μg/L)、高(40 μg/L)3个水平的加标回收试验。在同一自然日对高、低两个浓度的混合尿液进行6次平行测定,考察方法的日内精密度;在不同自然日分6次对相同的混合尿液样品进行测定,考察方法的日间精密度。结果如表4所示,8种目标分析物的加标回收率为91.1%~110.5%, RSD为1.2%~9.0%,日内精密度为2.9%~7.8%,日间精密度为6.2%~10%。

**表4:** 8种目标物的加标回收率、日内精密度和日间精密度(*n*=6)

Compound	Low concentration			Medium concentration			High concentration			Inter-day RSDs/%			Intra-day RSDs/%	
	Recovery/%	RSD/%		Recovery/%	RSD/%		Recovery/%	RSD/%		Low	High		Low	High
PNP	97.8	6.7		91.9	2.2		91.3	2.8		3.4	2.9		6.3	8.2
TCPY	102.2	4.2		101.2	1.7		93.1	1.6		3.6	3.7		9.1	7.8
3-PBA	92.5	8.6		97.1	1.2		97.6	2.2		5.3	3.6		7.6	9.3
4F-3PBA	93.5	9.3		99.1	2.5		99.7	1.8		6.0	3.0		7.1	8.8
cis-DCCA	99.6	5.4		100.5	3.4		98.8	2.5		6.8	7.8		8.7	9.3
trans-DCCA	96.9	9.0		98.4	2.1		96.4	3.8		4.7	3.6		6.2	8.9
2,4-D	105.7	2.4		110.5	3.1		107.8	1.2		3.4	3.7		8.8	10
2,4,5-T	91.1	7.0		94.7	1.6		95.6	1.8		2.9	3.9		7.4	9.8

### 2.6 实际样品测定

利用本方法测定了214份人群尿液样本,结果见表5。其中,除2,4,5-T未检出外,其余7种目标分析物均有检出,检出率为2.8%~99.1%。尿液中TCPY和PNP的检出浓度较高,中位值分别为2.0 μg/L和1.8 μg/L。3-PBA、*trans*-DCCA处于同一污染水平,中位值分别为0.81 μg/L和0.99 μg/L。上述结果提示农药对人群的暴露不容忽视。

**表5:** 人尿样品中8种目标物的含量

Compound	Detection rate/%	Mass concentrations/(μg/L)				
		Min	P_25_	Median	P_75_	Max
PNP	99.1	<MDLs	1.1	1.8	3.9	15
TCPY	98.1	<MDLs	0.95	2.0	4.8	52
3-PBA	94.4	<MDLs	0.51	0.81	1.8	22
4F-3PBA	2.80	<MDLs	<MDLs	<MDLs	<MDLs	0.39
cis-DCCA	63.1	<MDLs	<MDLs	0.44	0.72	5.3
trans-DCCA	99.1	<MDLs	0.50	0.99	2.6	44
2,4-D	94.4	<MDLs	0.20	0.35	0.55	6.0
2,4,5-T	0	<MDLs	<MDLs	<MDLs	<MDLs	<MDLs

P_25_ and P_75_: the 25th and 75th percentiles, respectively.

## 3 结论

本文利用96孔固相萃取,建立了尿中8种农药及农药代谢物的UPLC-MS/MS分析方法,系统评估了前处理方法、基质效应及方法学指标。方法采用96孔固相萃取高通量处理样品,处理效率高,同时兼具操作简单、定量准确、检出限低、重复性好的特点,适用于开展人尿中多种农药及农药代谢物的快速分析测定。
